# Reconstructing SARS-CoV-2 lineages from mixed wastewater sequencing data

**DOI:** 10.1038/s41598-024-70416-4

**Published:** 2024-08-31

**Authors:** Isaac Ellmen, Alyssa K. Overton, Jennifer J. Knapp, Delaney Nash, Hannifer Ho, Yemurayi Hungwe, Samran Prasla, Jozef I. Nissimov, Trevor C. Charles

**Affiliations:** 1 Department of Biology, University of Waterloo, Waterloo, ON Canada; 2 Metagenom Bio Life Science Inc., Waterloo, ON Canada; 3 Present Address: Department of Statistics, University of Oxford, Oxford, United Kingdom

**Keywords:** SARS-CoV-2, Wastewater, Sequencing, Variant of concern, Genome informatics, Next-generation sequencing, Viral genetics, SARS-CoV-2

## Abstract

Wastewater surveillance of SARS-CoV-2 has emerged as a critical tool for tracking the spread of COVID-19. In addition to estimating the relative case numbers using quantitative PCR, SARS-CoV-2 genomic RNA can be extracted from wastewater and sequenced. There are many existing techniques for using the sequenced RNA to determine the relative abundance of known lineages in a sample. However, it is very challenging to predict novel lineages from wastewater data due to its mixed composition and unreliable genomic coverage. In this work, we present a novel technique based on non-negative matrix factorization which is able to reconstruct lineage definitions by analyzing data from across different samples. We test the method both on synthetic and real wastewater sequencing data. We show that the technique is able to determine major lineages such as Omicron and Delta as well as sub-lineages such as BA.5.2.1. We provide a method for determining emerging lineages in wastewater without the need for genomic data from clinical samples. This could be used for routine monitoring of SARS-CoV-2 as well as other emerging viral pathogens in wastewater. Additionally, it may be used to determine more full-genome sequences for viruses with fewer available genomes.

## Introduction

SARS-CoV-2, the virus which causes COVID-19, has been continuously evolving into new lineages and sub-lineages since its discovery in humans in 2019^1–3^. New lineages and sub-lineages can differ in virulence, transmissibility, symptoms, and other factors that cause public health agencies to categorize them as variants of concern (VOCs)^3^. The genome of SARS-CoV-2 consists of genes in an order similar to previous coronaviruses: non-structural ORFs *ORF1a* and *ORF1b* followed by structural genes *spike (S), envelope (E), membrane (M),* and *nucleocapsid (N)*^2^. The spike protein encoded by the *S* gene allows interaction with the human angiotensin-converting enzyme 2 (ACE2) receptor for cell entry during infection and portions of this gene acquire mutations that are more likely to lead to immune escape^2–4^. The *E, M*, and *N* genes encode for packaging proteins^2^. Mutations do not always cause changes in the viral structure or behavior, but can be used as markers to identify sub-lineages. Lineages can be monitored using quantitative PCR (qPCR) or digital PCR (dPCR) assays specifically designed to target lineage-defining mutations, however, the number of mutations that can feasibly be targeted is limited by the short length of the genomic targets required in PCR experiments^1^. Additionally, development of each mutation-targeting PCR assay requires prior knowledge of lineage defining mutations^5^. Whole genome sequencing (WGS) provides an alternative approach that is not constrained by mutation targeting or prior knowledge of mutations, but is more time-consuming and expensive^6^. In practice, most WGS experiments in wastewater still require PCR amplification, in a series of overlapping amplicons, however these amplicons are typically designed to target more conserved sequences^7^. Whereas PCR primers often need to be redesigned for each new lineage, amplicon panels only need to be redesigned if there is a mutation in a primer-binding site which substantially reduces amplification of an amplicon. Clinical sampling provided the first sample material for both qPCR and whole genome sequencing of SARS-CoV-2^8^. A large number of clinical samples from tested individuals are required to follow trend data in a community. This limitation makes wastewater sequencing an attractive alternative as a less invasive way to survey whole communities with fewer samples^8^. Whole genome sequencing and qPCR can be performed on viral RNA extracted from wastewater samples for the purpose of variant tracking because SARS-CoV-2 is shed in feces^6,9,10^. When a new lineage is defined, the constellation of mutations that defines it can be searched for in the composite SARS-CoV-2 genomic sequences isolated from wastewater samples and be used to estimate the percentage of that population’s COVID-19 cases which are caused by each lineage^6^. Wastewater sequencing and analysis, known as wastewater-based surveillance (WBS), can capture the arrival of new variants in a community if they have previously been defined elsewhere using tools which identify lineages based on defined constellations such as Freyja, cojac, and Alcov^6,8,11–13^. Ideally, WBS could be used to track the emergence of variants which have been seen clinically in other parts of the world prior to or during early transmission to new regions. Many current WBS variant analysis tools rely on existing lineage definitions obtained by analyzing clinical sequencing data^14,15^. This delays the detection and monitoring of emerging variants. Therefore, it would be very useful if WBS analysis did not need to rely on existing lineage definitions to detect emerging variants in a community. Additionally, even known lineage definitions are usually not specific to regional variants, so the lineage composition predictions which rely on them are usually slightly inaccurate as they don’t capture regional mutations due to low prevalence globally^16,17^. In short, it is difficult to track the emergence of new variants in wastewater if we are only looking for variants which have already appeared in clinical samples. For instance, current methods would have been incapable of identifying an emerging variant such as Omicron until it was well-characterized by clinical sequencing. Finally, we would like to be able to use wastewater to study poorly characterized viruses such as emerging pathogens. Having the capability to use wastewater as a source of discovering all the current variants of a virus would provide useful context early in studying zoonotic pathogens, or even plant viruses. As with abundance estimation, the challenges for lineage discovery are two-fold: the data is usually composed of a mixture of different lineages and there are often substantial gaps in coverage. This means that taking a consensus genome from a wastewater sample will usually have large gaps (which are called to the reference) and is likely to contain mutations from multiple lineages especially if the sample isn’t strongly dominated by a single lineage. However, the frequencies of mutations which belong to a particular variant will be correlated within each sample and these correlations will be found across multiple samples which contain the same variant. Therefore, if we find mutations which are correlated over many samples we may deduce that they belong to the same lineage. This paper illustrates how such deductions can be used to reconstruct SARS-CoV-2 lineages in wastewater.

## Methods

### Code availability

The implementation of the method was written in Python. It has been tested on macOS and is available for download at https://github.com/Ellmen/derived-wastewater-lineages.

### Preprocessing the samples

Wastewater samples were collected as part of the Wastewater Surveillance Initiative from sites across Ontario (Canada). Wastewater sample viral content was concentrated using Nanotrap$\circledR$ Microbiome A Particles (Ceres Nanosciences, Inc., 44202). RNA extraction was automated on a QIAcube Connect using QIAGEN RNeasy mini kit (QIAGEN, 74116, 9002864). The optional, on-column DNase II treatment step was not performed during RNA extraction. RNA was reverse transcribed to yield cDNA using the LunaScript$\circledR$ RT SuperMix Kit, according to the manufacturer’s protocol (New England Biolabs, M3010L). SARS-CoV-2 cDNA was then amplified by PCR using the ARTIC V4.1 primers (IDT, 10011442) and Q5 High Fidelity 2X Master Mix (NEB, M0492L) according to the nCoV-2019 sequencing protocol v3 provided by IDT. ARTIC V4.1 primers are designed to amplify 98 400 bp amplicons which must be re-assembled during analysis to complete the SARS-CoV-2 genome^7^. After PCR, a 0.8X bead to sample ratio of AMPure XP (Beckman Coulter, Inc., A63881) beads were used for sample cleanup and PCR fragments were prepared for sequencing using the Illumina DNA Prep kit (20060059) during which a size of 350-400 bp was selected. All samples were sequenced using an Illumina MiSeq using 2x250 reads and V2 chemistry (MS-102-2003).

The resulting read fastq data was processed by Gromstole1.0^18^ which runs cutadapt^19^ and minimap2^20^ to trim and map the reads to a reference. Gromstole determines the frequency of each observed mutation (i.e., difference from the reference sequence) in a sample and saves the mutation frequencies, and positional depth of coverage in CSV files. We parsed the CSVs to determine the set of all mutations which were observed at least once with a depth of at least 20 reads. Empirically, the 20 read cutoff prevented the inclusion of mutations which were the result of sequencing errors or contamination. Samples were then encoded as vectors wherein each element corresponds to the observed frequency of a given mutation in that sample.

### Imputing data from amplicon dropouts

All of our data vectors must have the same dimension, so we must have a value for the frequency of each mutation. This is sometimes problematic because we often get dropout of amplicons, due to a lack of PCR amplification over certain genomic regions, and as a result do not have information about the mutations located within dropped amplicons. To solve this, we impute missing data using a k-nearest neighbours (KNN) model. KNN imputation has been used in a similar setting to fill in gaps in DNA micro-array data^21^. KNN finds the *k* most similar data points (samples) and fills in the missing mutations with the average of their frequencies. This means that the missing mutation frequencies have a tendency to be filled in with values from samples with similar lineage abundances. We used the standard scikit-learn^22^ implementation of KNN imputation with its default value of $k=5$. Imputing from the 5 nearest neighbours was a reasonable balance between having enough similar samples without imputing from samples which were too dissimilar.

### Non-negative matrix factorization

As discussed, we cannot assume that each variant will be present at the consensus level. Additionally, the frequencies of mutations in a sample are highly variable, so each sample may not show an accurate snapshot of the lineages it contains. However, we know that on average, the frequency of a given mutation in our sample will be the sum of the abundances of the lineages which contain that mutation. This means that frequencies of mutations which are contained in the same lineage will be correlated across different samples. Our task then is to determine which mutation frequencies tend to be correlated (increase and decrease together) across all samples, and predict that those mutations form a lineage.

To solve this problem, we use a technique called non-negative matrix factorization (NMF) which is similar to PCA.

NMF has been used to find similar types of patterns in image data and facial recognition programs^24^.

NMF optimizes the loss function in Eq. (1):

1 $$\begin{aligned} L(W,H) = 0.5*||X - WH||^{2}_{Fro} \end{aligned}$$

Where $||X||^2_{Fro} = \sum _{i,j}A_{ij}^2$ is the Frobenius norm of a matrix and the optimization is subject to the additional constraint that all entries of *W* and *H* be non-negative. In our task, *X* corresponds to the observed mutation frequencies within each sample. Then, we seek to find matrices *W* and *H* which correspond to lineage frequencies per sample, and mutation frequencies per lineage such that their product is as close as possible to *X*. If there are *m* idenitified mutations, *s* samples, and *n* lineages then *X* is $s{\times }m$, *W* is $s{\times }n$, and *H* is $n{\times }m$. The rows of *H* are also called the components, and the number of components, *n* is a parameter of the model. Since positive linear combinations of the components of *H* are used to approximate the observed mutation frequencies, each component corresponds to a lineage where the non-zero elements are mutations which are present in that predicted lineage. Similarly, the non-negative elements of *W* correspond to the predicted frequency of each lineage in each sample. Crucially, with enough data, we can predict the lineages and frequencies without any prior knowledge of real variants.

NMF is conceptually similar to PCA with two key differences. The first difference is that the learned values are all positive which is important since lineage frequencies and mutation frequencies are positive. The second difference is that the learned components need not be orthogonal. This allows us to learn similar lineages with overlapping mutations, such as subvariants. NMF can also be trained to minimize the $\ell _1$ norm of the learned matrices which encourages sparseness. This is desirable since the vectors correspond to lineage definitions. Since there are tens of thousands of possible mutations and each lineage only contains tens to hundreds, the lineage definitions should be sparse^26^, however we found the learned solutions were sparse without the need for regularization.

We used the scikit-learn implementation of NMF which minimizes the $l_2$ error using the coordinate descent algorithm described in^27^.

### A framework for finding conserved lineages

**Figure 1:**
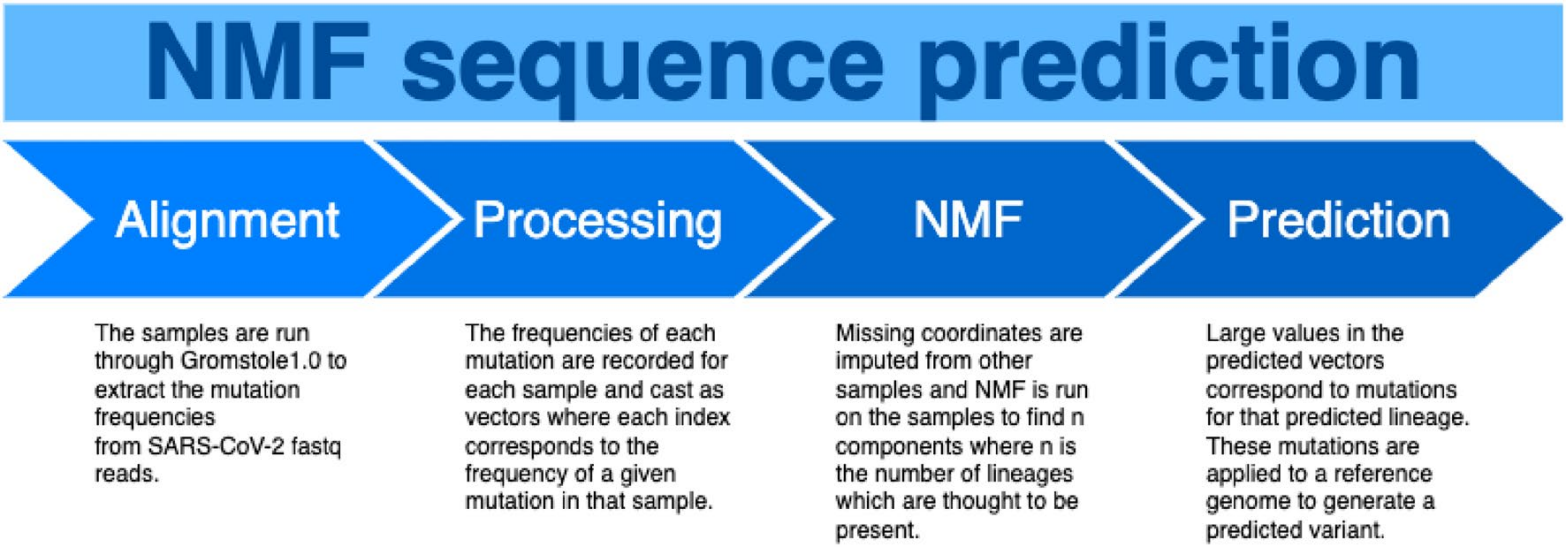
Overview of our proposed method for determining viral variant genomes from wastewater samples.

The strategy we devised for extracting lineage definitions from our wastewater samples is illustrated in Fig. 1 and described as follows:

1. The samples are run through Gromstole1.0 to extract the mutation frequencies from SARS-CoV-2 fastq reads.
2. The frequencies of each mutation are recorded for each sample, where mutations with a coverage of less than 20 reads are omitted.
3. The samples are cast as vectors where each entry corresponds to the frequency of each of the observed mutations.
4. Missing data in each of the sample vectors is imputed using KNN imputation with $k=5$.
5. NMF is run on the samples to find *n* components where *n* is the number of lineages which are thought to be present.
6. The learned NMF vectors are normalized (divided by the maximum value) so that the highest value of each is 1.
7. For each lineage vector, all mutations which have a corresponding value of at least 0.25 are included in that lineage’s definition. The 0.25 cutoff was chosen instead of 0.5 to be sensitive enough to capture mutations even when some samples are incorrectly imputed to 0.
8. The mutations are applied to the reference genome of SARS-CoV-2 to create fasta files which can be fed into downstream phylogenetic analysis tools to classify which lineage/sub-lineage they represent.

## Results

### Finding lineages in simulated reads

Synthetically combined datasets with known frequencies are a valuable control and self-test when developing wastewater data analysis techniques. One such dataset has been created to test frequency prediction tools by^28^, available on Github (https://github.com/sgsutcliffe/ww_benchmark), which we used to test our method’s ability to detect known lineages.

The dataset contained simulated reads from 35 genomes, representing four major SARS-CoV-2 lineages (BA.1, BA.2, Delta, and a “deltacron” recombinant lineage) as well as a synthetic SARS-CoV-2 lineage which contained random mutations. The dataset was composed of 100 simulated samples with different combinations of the five lineages and some with simulated amplicon dropout. The proportion of each lineage in a sample ranged from as little as 1% to 100%.

We ran the method on the 100 simulated samples and identified 5 NMF components (corresponding to the five lineage definitions included in the dataset). Note that this tool requires the user to input the number of lineages to identify in the sample. The five predicted lineages (NMF components) were run through Nextclade^29^, a tool that performs sequence alignment, mutation calling, and clade assignment for various pathogens including SARS-CoV-2, as Pangloin struggles with recombinant lineages making it unsuitable for this analysis. As expected, Nextclade classified the five predicted lineages as BA.1.18 (BA.1), AY.4 (Delta), BA.2.3 (BA.2), B (undetermined synthetic), and XS (Deltacron).

**Figure 2:**
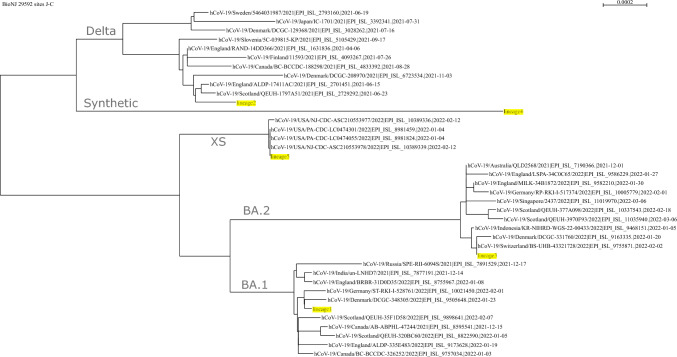
Phylogenetic tree (NJ) showing how the predicted lineages cluster with the isolates that were used to simulate the reads. Predicted lineages are highlighted in yellow. Predicted lineage 4 clusters alone, and is likely the dataset’s novel synthetic lineage.

Accession numbers for each of the 34 genomes which were used to simulate the reads are available on GitHub (https://github.com/sgsutcliffe/ww_benchmark/blob/main/consensus_lineages.txt). Each of these sequences were downloaded from GISAID and classified with Nextclade, alongside the five predicted sequences from our method. Nextclade predicted a range of BA.1, BA.2, and Delta sub-lineages. All deltacrons were predicted to be XS which corresponded with our predicted deltacron sequence. The synthetic genome was not included since it was not uploaded to GISAID. We downloaded the alignment from Nextclade and built a neighbour joining (NJ) tree using Seaview^30^, shown in Fig. 2. Our predicted lineages are highlighted in yellow and clearly cluster with the four major lineages. Additionally, our fourth predicted lineage, “lineage4” clusters distinctly alone, which would be consistent with a synthetic genome containing random mutations. Therefore, our method was able to pick out the four real major lineages in the simulated dataset, as well as the novel synthetic lineage.

### Finding major VOCs across all samples

Ongoing wastewater collection for surveillance from sites across Ontario (Canada) provided an environmental dataset of raw SARS-CoV-2 amplicon sequencing data. All available data at the time of download (1026 samples collected between October 2021 and June 2022) from our routine sequencing of Ontario wastewater was processed using NMF with 3 components so that 3 lineages would be predicted. We applied the mutations listed in the predicted lineages to the SARS-CoV-2 reference genome to create a fasta with the sequence for each NMF-predicted lineage. We ran Pangolin^31^ on the resulting fastas to assign a lineage to each of them. Pangolin also runs a tool called Scorpio which assigns lineages and provides a confidence score for the particular lineage call. The predicted lineages for Pangolin and Scorpio along with the Scorpio support values are shown in Table 1.

**Table 1:** Lineage assignments for each of the NMF-predicted lineages from all samples. The predicted lineages were all highly abundant in Ontario when these samples were collected^32^.

Isolate	Lineage	Scorpio call	Scorpio support
Lineage1	BA.2	Omicron (BA.2-like)	0.97
Lineage2	BA.1.1	Omicron (BA.1-like)	0.88
Lineage3	B.1.617.2	Delta (B.1.617.2-like)	0.92

Both Pangolin and Scorpio agree on all three and Scorpio indicates strong support for the predicted lineages (Pangolin does not provide support values)

The three NMF-predicted lineages were BA.2, BA.1.1, and B.1.617.2. All three of these were highly abundant in clinical sequencing data in Ontario during the time frame that we analyzed. B.1.617.2 is the parent lineage for all delta sub-lineages which were dominant in Ontario before being replaced by Omicron (BA.1.1). Eventually BA.2, another Omicron sub-lineage, replaced BA.1.1^32^. Together, these give an accurate snapshot of the most significant lineages in Ontario between October 2021 and June 2022.

**Figure 3:**
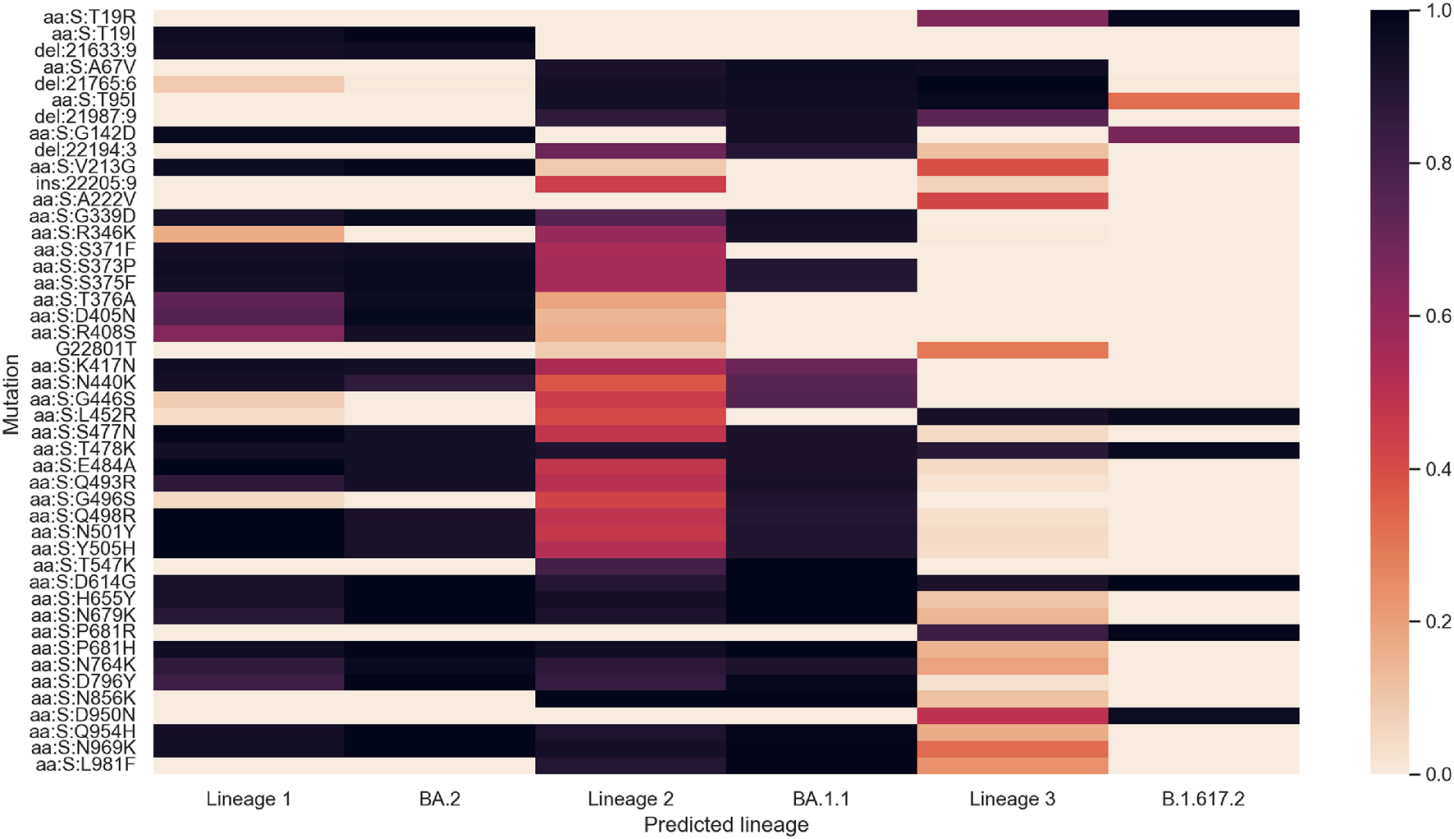
Heatmap showing the learned spike mutation values of the predicted lineages next to the frequency with which those mutations are observed in the corresponding lineage according to outbreak.info. Nonsynonymous mutations which cause the same amino acid change are grouped together and labelled by the amino acid change.

We downloaded the frequency of each mutation for the three predicted lineages from outbreak.info and compared them to the learned mutation values^33^. Figure 3 shows the values for the spike mutations next to the frequency with which those values are observed in clinical sequences. For NMF-predicted lineages, the value is the normalized value from that lineage’s learned NMF component. For known lineages, the value is the proportion of known clinical genomes which contain that mutation. In general, the predicted lineages agree well with their analogous known lineages. The lineages on outbreak.info do not include synonymous mutations or insertions. Some mutations may represent legitimate local variation (like S:A222V), albeit with poor coverage and therefore a small sample size. The confidence and accuracy of the predictions decreases with each consecutive lineage which is logical because the components in NMF are ranked according to their relative importance.

**Figure 4:**
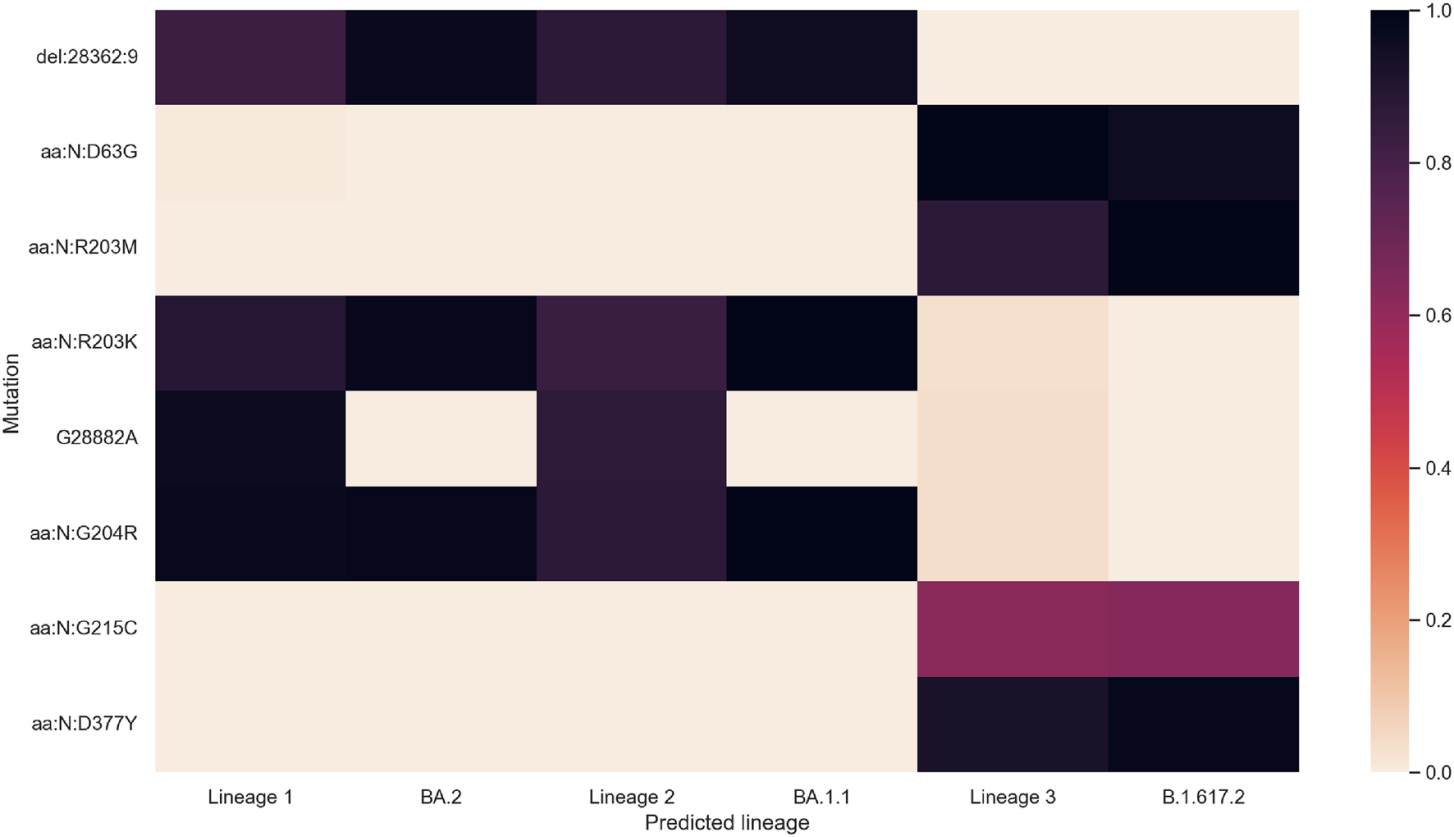
Heatmap showing the learned N gene mutation values of the predicted lineages next to the frequency with which those mutations are observed in the corresponding lineage according to outbreak.info. Nonsynonymous mutations which cause the same amino acid change are grouped together and labelled by the amino acid change.

Figure 4 plots the values for the N gene. All mutations which are predicted to be significant agree with the outbreak.info data, including the variable presence of N:G215C in Delta. The *N* gene carries fewer mutations than the *S* gene and has much better coverage which probably leads to increased accuracy in the predicted lineages.

### Finding SARS-CoV-2 sub-lineages in a single run

We also ran the NMF method to look for two lineages in a single sequencing run with samples from across Ontario in late June 2022. The lineage predictions are shown in Table 2.

**Table 2:** Lineage assignments for each of the NMF-predicted lineages from a single run in late June 2022.

Isolate	Lineage	Scorpio call	Scorpio support
Lineage1	BA.5.2.1	Omicron (BA.5-like)	0.97
Lineage2	BA.2.12.1	Omicron (BA.2-like)	0.97

The method accurately predicts BA.5 and BA.2 including sub-lineages which have been found in clinical samples with very high scorpio support.

Using samples from a single run, the method was able to accurately predict the two major Omicron lineages in Ontario at the time, BA.2 and BA.5. Surprisingly, the method was able to pick up all mutations with enough accuracy to predict specific sub-lineages of the two. Both of these sub-lineages have been identified in Ontario at the time according to outbreak.info, although the prevalence of BA.5.2.1 in clinical cases is lower than the prevalence predicted in wastewater using known lineage prediction pipelines (i.e., Alcov)^11,34^.

**Figure 5:**
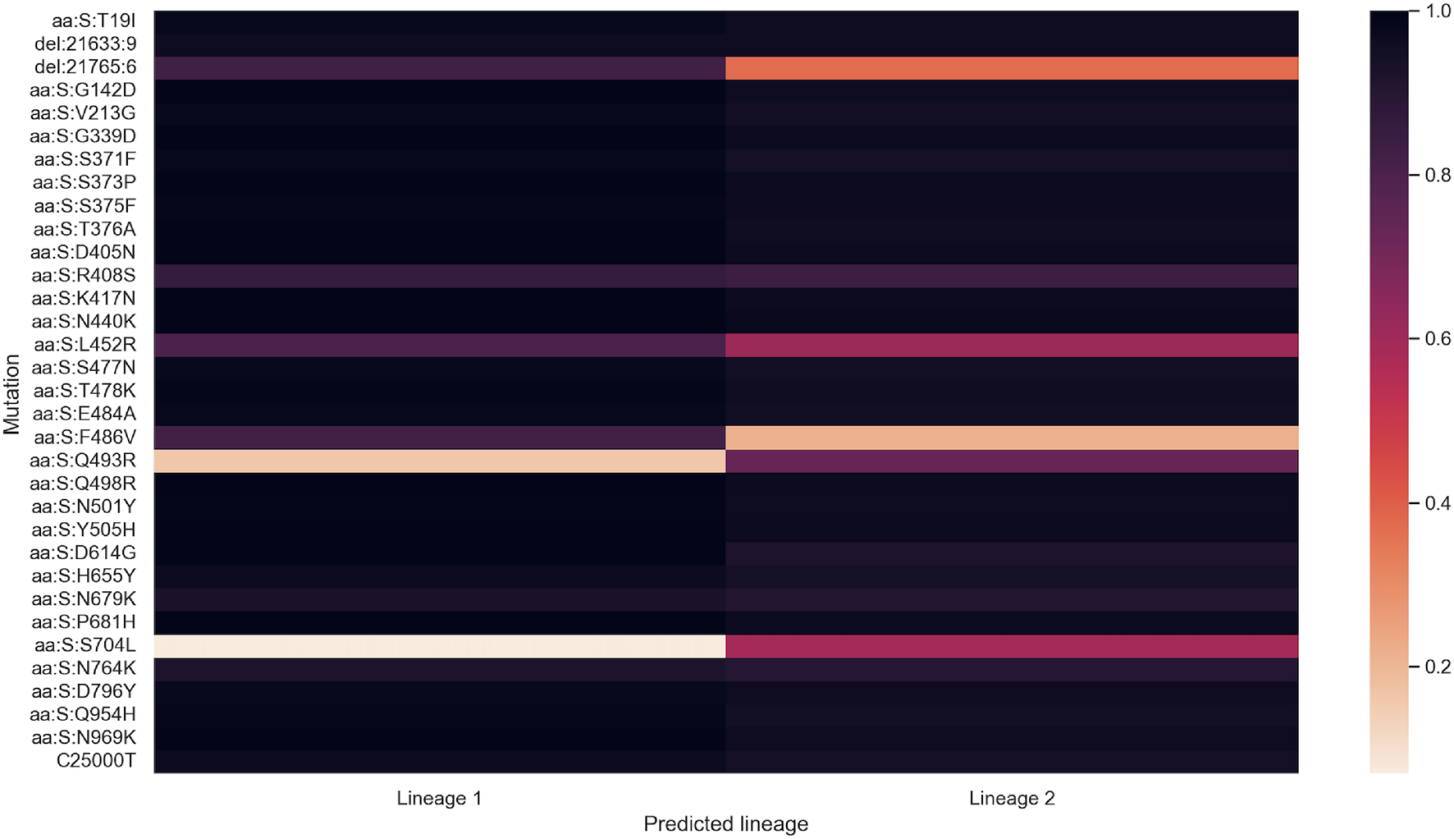
Heatmap showing the learned spike mutation values of the predicted lineages in the single run. Nonsynonymous mutations which cause the same amino acid change are grouped together and labelled by the amino acid change.

We plotted the predicted values of the spike mutations for the two sub-lineages, which are shown in Fig. 5. BA.2 and BA.5 are very similar which can pose a challenge for the method but it was able to identify distinguishing mutations such as S:F486V.

It is worth noting that sub-lineages are notoriously difficult to distinguish in wastewater, even when mutations are known. This is because closely-related sub-lineages are usually only differentiated by a few mutations and the frequency of those mutations can vary widely from sample to sample. Our primary aim was to develop a method which is capable of discovering SARS-CoV-2 lineages and mutations without the need for clinical sampling or WBS. Surprisingly, we are able to identify the mutations with such accuracy that not only can we deduce the major lineages which are present in a sample, but also accurately identify the specific sub-lineages which are most abundant without the need for lineage definitions. The accuracy likely comes from the ability of the method to pool information from multiple samples which works to smooth and reduce some of the noise within individual samples.

## Discussion

Here, we present a new method for determining the RNA sequences of SARS-CoV-2 variants from wastewater data, without prior lineage definitions, which could be used in surveillance programs. The method uses NMF to deconvolute the mixed data into single lineages. We show that the method is capable of reconstructing real SARS-CoV-2 lineages down to the sub-lineage level from mixed samples, as demonstrated on experimental and synthetic datasets.

We provide a starting point for using wastewater sequencing data to determine lineages without relying on clinical sequencing for lineage definitions, however some technical challenges remain. In practice, the number of lineages circulating in a given region has been low for much of the SARS-CoV-2 pandemic, but it would be useful to automatically determine this number, rather than needing to input a value. If the number were automatically determined this would also be useful in deconvoluting closely related sub-lineages such as those seen more recently with BQ.1* and XBB*^29^. Currently, the number can be adjusted based on the desired resolution. For instance if only parental lineages are required a low number should be used, whereas closely related sub-lineages can be predicted by setting this number higher. Additionally, the imputation of missing values likely introduces error into the lineage predictions. It may be possible to modify the optimizer during the matrix factorization to simply ignore missing values in the distance calculation, removing the need for imputation.

In contraast to lineage identification tools, none of the lineages predicted in by our method were previously known to the model^11–13^. The ability to predict lineages without constellations or definitions of those lineages would allow lineages to be identified before a sufficient number of clinical sequences has been sequenced to define a new lineage. In this study existing data was used to verify that any predicted lineages defined by the model can be trusted, by showing that they align with true SARS-CoV-2 lineages from the clinical and wastewater data at that time. Additionally, the predicted presence of the novel synthetic genome from the bench-marking dataset demonstrates the model’s ability to predict lineages for which there are no clinical genomes. The NMF-predicted lineages from the methods described in this study could be manually curated and used to propose lineage definitions after clinical sequencing of SARS-CoV-2 becomes infrequent or before clinical sequencing programs are established in future pandemics.

While SARS-CoV-2 wastewater sequencing has been of great interest during the COVID-19 pandemic, there are many other viruses which could benefit from this method. For instance, our method could be used to determine lineages for understudied plant viruses or seasonal influenza and RSV outbreaks. A similar method may be of interest outside of wastewater contexts, such as epigenetic sequencing of human cell cultures, or sequencing mixed viral populations within a single patient. Our method is quite flexible, requiring only a reference genome, the locations of genes on the reference for naming mutations, and the desired number of lineages to predict.

## Conclusion

Herein, we present a method for determining viral lineage sequences from mixed samples in wastewater. Whereas much existing literature aims to determine the abundance of known lineages, our method enables the determination of novel lineages. We show the efficacy of the method both on synthetic and real SARS-CoV-2 wastewater sequencing data. On the synthetic dataset, the inferred genomes cluster appropriately with the genomes used to generate the data. On the real data, the inferred genomes match known lineages which were circulating in Ontario while the data was collected. Together, this provides a method for determining emerging lineages from wastewater without the need for existing clinical sequences. It may also be used to determine lineages of less-studied viruses such as plant viruses in wastewater.

### Supplementary Information

Supplementary Information.

## Data Availability

The raw wastewater sequencing data which was used as a test dataset is available at bioproject PRJNA1027858 on the Sequence Read Archive https://www.ncbi.nlm.nih.gov/bioproject/PRJNA1027858.
