# Supplementary material for: Reconstructing SARS-CoV-2 lineages from mixed wastewater sequencing data
Source: Sci Rep. 2024 Aug 31;14:20273. doi: 10.1038/s41598-024-70416-4 (PMC11365997; doi:10.1038/s41598-024-70416-4)
Supplement: Supplementary file 1 — Supplementary Information. [file 41598_2024_70416_MOESM1_ESM.pdf]

Supplementary information for Learning novel SARS-CoV-2 lineages from wastewater sequencing data

Isaac Ellmen, Alyssa K. Overton, Jennifer J. Knapp, Delaney Nash, Hannifer Ho, Yemurayi Hungwe, Samran Prasla, Jozef I. Nissimov, Trevor C. Charles

June 2024

1 Reconstruction loss for different numbers of components

The number of components is a key parameter of our model since changing the number may change all of the components. Having too many components can cause the model to split single lineages into component parts or generate partial lineages which represent the difference between related lineages. Having too few can cause the model to combine lineages or miss lesser expressed lineages. In most cases, this number can be selected rationally, for instance there are usually only two lineages circulating at a given time. We investigated the relationship between the reconstruction loss, which is similar to the explained variance ratio in PCA, for different numbers of components. Figure 1 shows the number of components and the reconstruction error.

In contrast to PCA, the model needs to be retrained for each value of n, however most of our models trained within seconds. Also in contrast to explained variance ratio, there is not a known minimum loss which is realistic for the model to achieve. The loss tails off slightly after the "correct" number of lineages has been reached, however the effect is subtle. In practice, inspecting the sequences for different numbers of components is likely the most reliable approach at this time.

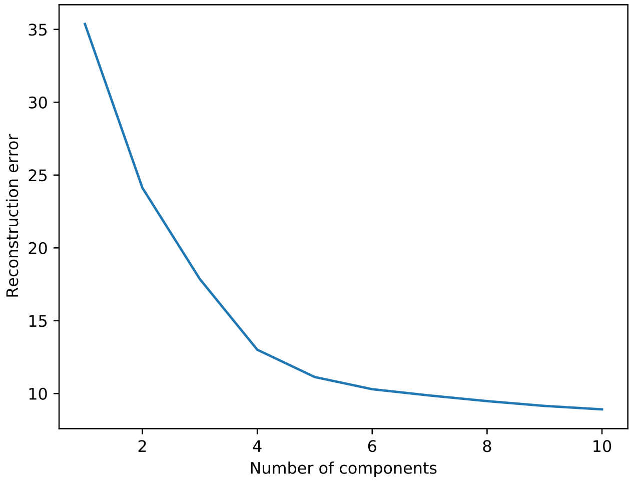

(a) Synthetic lineages

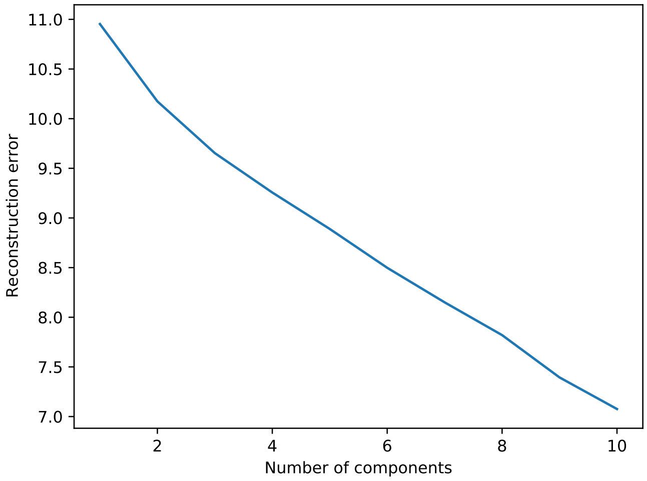

(b) Single run from June 2022

Figure 1: Number of lineages vs. reconstruction error for two datasets
